# Quasi-Static Compression and Low-Velocity Impact Behavior of Tri-Axial Bio-Composite Structural Panels Using a Spherical Head

**DOI:** 10.3390/ma10020185

**Published:** 2017-02-15

**Authors:** Jinghao Li, John F Hunt, Shaoqin Gong, Zhiyong Cai

**Affiliations:** 1 USDA Forest Service, Forest Products Laboratory, Madison, WI 53726, USA; jli@fs.fed.us; 2 Department of Biomedical Engineering, Wisconsin Institutes for Discovery, and Materials Science and Engineering, University of Wisconsin-Madison, Madison, WI 53715, USA; Shaoqingong@wisc.edu

**Keywords:** low-velocity impact, quasi-static compression, bio-composite laminate material, energy absorption, carbon fiber fabric faces, foam core, isogrid core

## Abstract

This paper presents experimental results of both quasi-static compression and low-velocity impact behavior for tri-axial bio-composite structural panels using a spherical load head. Panels were made having different core and face configurations. The results showed that panels made having either carbon fiber fabric composite faces or a foam-filled core had significantly improved impact and compressive performance over panels without either. Different localized impact responses were observed based on the location of the compression or impact relative to the tri-axial structural core; the core with a smaller structural element had better impact performance. Furthermore, during the early contact phase for both quasi-static compression and low-velocity impact tests, the panels with the same configuration had similar load-displacement responses. The experimental results show basic compression data could be used for the future design and optimization of tri-axial bio-composite structural panels for potential impact applications.

## 1. Introduction

**Figure 1:**
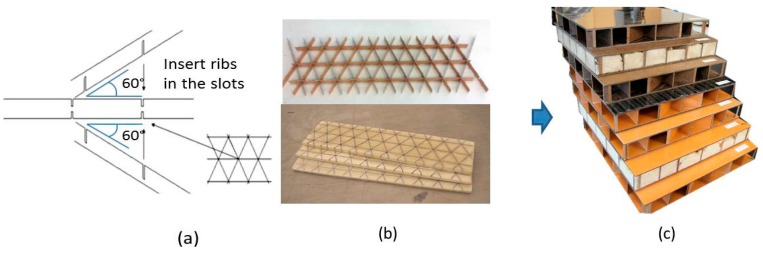
Various designs and configurations for tri-axial engineered bio-composite panels, (**a**) interlocked tri-axial structure; (**b**) structural core without foam or with foam and (**c**) tri-axial composite panels with various configurations.

Structural composite panels are generally assembled using two stiff faces bonded to a lightweight structural core. Specific configurations are engineered for strength, stiffness, or energy absorption characteristics for many applications used in buildings, transportation, aircraft, marine, etc. [1,2,3,4]. There are many core structural options (e.g., honeycomb, foam, corrugated materials, trusses, grids, lattices, etc.). Each type has its own unique characteristics that can be used to provide optimum performance for different applications. Recently, an efficient interlocking grid system has been developed. It has one set of linear ribs with single slots from one edge for part of the rib’s width, for the other set of linear ribs, they have single or double slots from one or both edges to create an interlocking grid system (Figure 1) [5]. With this system, it is possible to modify the construction to obtain various grid patterns such as square, rectangular, triangular, hexagonal, or mixed structures [6]. As the number of linear rib directions is increased, core shear stress resistance is improved for multidirectional loadings. Researchers have shown that panels with structural core ribs aligned 60 degrees apart had better mechanical performance than a honeycomb structure [7]. However, there are few studies regarding impact damage investigations, so additional analyses are needed to continue the development of this type of structure using isogrid core sandwich structures.

Low-velocity impact can cause different types of damage and significantly affect the carrying capacity of structures. For example, in the aircraft industry, impact strength/resistance of exposed components must be sufficiently robust to prevent catastrophic failure of the structure [8]. Most of the previous low-velocity impact research has focused on foam or honeycomb structural panels. Schubel et al. performed low-velocity impact tests on sandwich structures with carbon fabric/epoxy laminates faces and polyurethane foam core material [9]. They analyzed the impact dynamic behavior using analytical and finite element model analyses to determine their effectiveness in predicting the impact behavior of the sandwich panel. Wang investigated the impact behavior and energy absorption of honeycomb sandwich panels [10]. The honeycomb cell-wall thickness and length, core thickness, and materials were all input factors and analyzed for their influence on panel performance. The results showed the thickness and length of honeycomb cell-wall had a significant effect on its impact behavior. However, different types of structures exhibited different impact behaviors, thus the results were limited to the specific structural panel being studied. Recently, the isogrid-stiffened/syntactic-foam core sandwich structure has been investigated by Li et al. [11]. The results showed that the design and configuration significantly influenced the impact behavior, and the isogrid triangular cells within the foam showed better impact capacity than just the laminated composite structure without foam. Additionally, the raw material and structural configuration of structural composite panels had significant effects on the impact behavior and damage [9,10,11]. However, there is no literature where the isogrid structural composites were made from bio-based material with an interlocking isogrid structure. 

At the USDA, Forest Products Laboratory (FPL), new research is being conducted to develop bio-composite materials to enhance performance for various engineering applications including tactical shelters, packaging, building materials, and air pallets [6,12,13,14,15,16,17,18,19,20,21,22]. High stiffness characteristics were the primary consideration for performance. A phenolic laminated paper made from wood fibers was used for the tri-axial interlocked core and faces of a sandwich panel. In the initial study, both static and dynamic mechanical behavior for these tri-axial wood-fiber-based composite panels was investigated using flatwise and edgewise compression tests, four-point bending static, and fatigue tests [6,12,13]. The results showed that these structural panels have the potential to provide good mechanical performance for various engineering applications. However, impact resistance, which has not been investigated, is considered a critical design parameter for some applications.

This paper investigates the impact behavior of wood-fiber-based tri-axial core composite sandwich panels with different core/face configurations using quasi-static compression tests and low-velocity impact tests. The energy absorptions and contact loads of panels were analyzed through different materials, design configurations, and test parameters.

## 2. Experimental Section

### 2.1. Material Properties

Phenolic impregnated laminated paper sheet (NP610, Norplex-Micarta Inc., Postville, IA, USA) was used for both core and faces. The laminated paper had a nominal paper: resin weight ratio of 75:25. The mechanical properties of the laminates were obtained according to ASTM test methods D638 and D695 [23,24]. The laminated paper has orthotropic properties with the primary strength directions designated machine direction (MD) and cross-machine direction (CD).

To enhance face stiffness and strength, a tri-axially woven carbon fiber fabric (QISO, A&P Technology, San Jose, CA, USA) was bonded to both outside faces of panels using an epoxy resin (U.S. Composites No. 635, West Palm Beach, FL, USA). The ratio of epoxy to hardener was 3:1. The bonded carbon fiber fabric and laminated paper were tested as a combined composite material for the faces. This composite face material was tested along the machine direction (MD) and cross-machine direction (CD).

The epoxy resin properties were obtained by forming a dog-bone test coupon and tensile tested. The shear strength of the epoxy resin was tested using a single lap joint of two laminated paper sheets.

Foam used to fill the void volume between the core ribs was self-expanding 3 lb density urethane foam (US Composites Inc., West Palm Beach, FL, USA). Materials properties for the tri-axial structural panel components are shown in Table 1.

### 2.2. Panel Design and Fabrication

A set of panels was fabricated with various interlocked tri-axial core configurations using 2.4 mm thick laminate material for the core and faces. Overall, the core thickness was 51 mm for all panels; the total nominal thickness of panels without or with carbon fiber fabric was 56 mm and 59 mm, respectively. For groups 1 to 6, the isogrid core spacing was 25.4 mm. The slot widths were cut to accommodate for the 60-degree angle at the rib configurations (Figure 1). For groups 7 to 13, 50.8 mm core grid was used. Two panel configurations (2 and 5) had expanding foam between the ribs (Figure 1b). Two panel configurations (3 and 6) had carbon fiber fabric bonded to the face laminates. Quasi-static compression was used on groups 1 to 3 and low-velocity impact tests were used on groups 4 to 13. Each group had three replicates. The testing plan and mean density for the panels are shown in Table 2. 

### 2.3. Quasi-Static Compression Test

**Figure 2:**
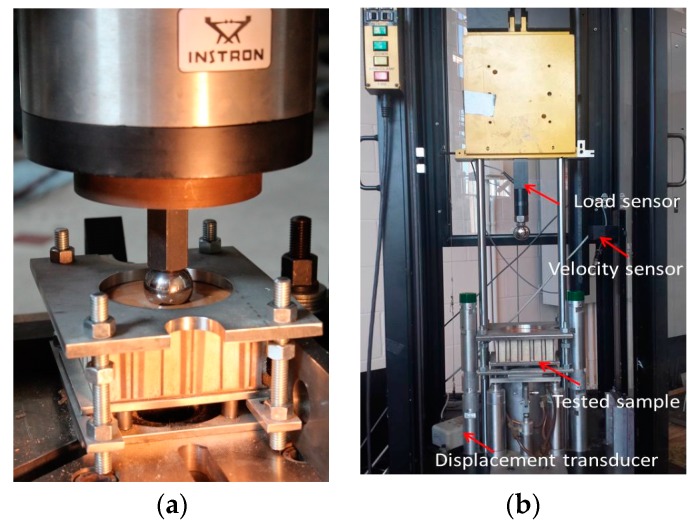
Testing set-up: (**a**) Quasi-Static compression; (**b**) Low-velocity impact

Three different panel configurations were used for initial quasi-static compression tests. These were used for comparison with the impact tests. The set-up for the quasi-static compression test for groups 1 to 3 is shown in Figure 2a. The test was modified from ASTM C365 [25], where the load was applied through a 38 mm diameter spherical head at the center of the triangular core element instead of a flat surface. The same clamping set-up that was used for the quasi-static compression tests was also used for the low-velocity impact tests. In order to make full use of loading table of the impact machine and minimize the localized effect of structural panel, the clamping fixture had a 102 mm diameter hole. Within the hole circumference included more than six full triangular lattice elements in the core of panel for both compression and impact tests. To verify the repeatability, three replicates were performed for each panel configuration. The cross-head compression rate was 10 mm/min. The dimensions for the panels were 127 mm × 127 mm. 

### 2.4. Low-Velocity Impact Test

Impact tests are generally divided into low-velocity and high-velocity evaluations. Each one elicits a different structural response and structural damage. For panels used for building construction, transportation or packaging products, low-velocity impact damage is a more common occurrence. To analyze this damage, different energy levels were employed in the low-velocity impact test. A Dynatup drop tower, model 8250, with a free-falling mass was used for the impact test following ASTM D7136 [26], Figure 2b. Three different energy levels 40 J, 80 J, and 120 J were used corresponding to drop weights of 22.5 kg at 360 mm and 170 mm and 33.3 kg at 360 mm producing impact velocities ranging from 2 to 3 m/s. In addition, three different diameter spherical load heads, 13 mm, 25 mm and 38 mm were used for our tests. The clamping fixture used to hold the impact test samples during impact had a 102 mm diameter opening. The sample was securely clamped by air cylinders. A 22.2 kN load cell was connected to the load head to measure the impact load. Impulse software was used to record and display load, impact speed, and displacement data. 

## 3. Results and Discussion

### 3.1. Quasi-Static Compression Test

**Figure 3:**
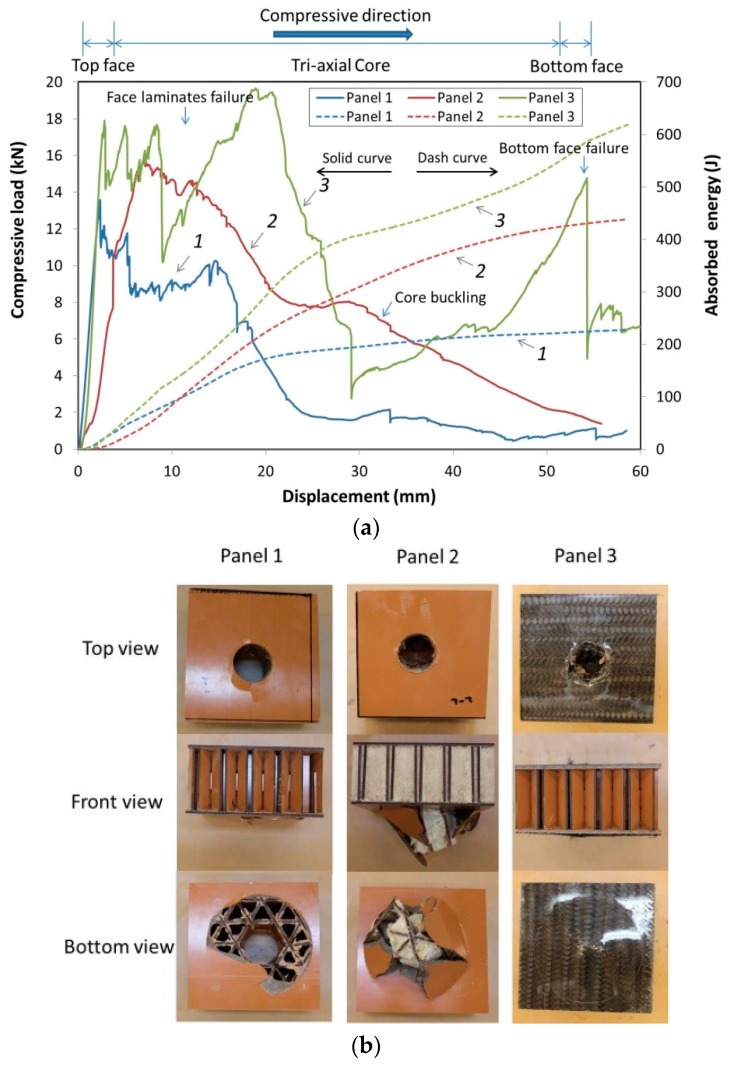
(**a**) Typical load and absorbed energy vs. displacement curves of panels in the quasi-static compression; (**b**) Typical failure modes of panels in the quasi-static compression.

The typical test curves are shown in Figure 3a for compressive load and accumulated energy vs. displacement. The solid curves show the typical compressive load vs. displacement for panels 1, 2, and 3, respectively. The displacement transducer recorded displacement data starting when the load-head contacted the top face of the panels. For this initial compression test, the load-head was positioned directly above the center of a triangular element in the core. As expected, the results showed the three different panel configurations had different quasi-static responses as shown in Figure 3. The compressive load for panel 1, laminated paper only, showed the load sharply increased as a function of displacement before the initial top face failure and then gradually decreased as the load-head penetrated into the core with only small fluctuations. There was no second load peak observed as the load-head passed through the bottom face of the panel. We believe this happened because when the load-head contacted the top face, the load transferred immediately through the ribs to the bottom face causing the bottom face to simultaneously fail before the load-head penetrated to the bottom face. For panel 2, with foam filled core, the load increased to a maximum compressive load as a function of displacement before the top face failed, and then the load gradually decreased until the load-head went through the top face and into the core. The foam core absorbed slightly more energy. The foam also helped stabilize the ribs, resisting core rib buckling. Thus, the maximum compressive load was higher than that of panel 1. For panel 3, with carbon fiber fabric faces and no foam filled core, the results were the highest maximum compressive load than the other two panel configurations. The carbon fabric provided increased stiffness when combined with the laminated paper faces. Furthermore, the compressive load-displacement curve had multiple peaks as the load-head passed through panel 3, Figure 3a. 

The dashed curves (Figure 3a) show typical accumulated energy absorption vs. displacement for panels 1, 2, and 3, respectively. The energy absorption was calculated by integrating the load and displacement curves. Panels 2 and 3 had higher energy absorption thus indicating that foam and carbon fabric significantly influenced the energy absorption. Panel 3, absorbed an average 670 J of energy, which was 80% higher than average for panel 2 at 373 J with foam filled core and was 213% higher than panel 1 which absorbed an average of 214 J. Panel 2, with foam filled core, had 74% higher accumulated energy absorption than panel 1. Comparing the average specific energy absorption (energy/density), Panel 3 was 1.42, which was 103% higher than panel 2 at 0.70 with foam filled core and was 202% higher than panel 1 at 0.47. The carbon fiber fabric reinforced face component was the significantly factor to improve the compressive performance of the panels.

Figure 3b shows the typical failure modes of panels 1, 2, and 3 with different panel configurations. All panels had similar damage on the top face; however, different failures were observed on the bottom, Figure 3b. The foam-filled core, panel 2, had the best integrity where the load had been dispersed into the structure assisted by the foam as compared with panel 1 that had more localized damage. For panel 3, the carbon fiber fabric that increased stiffness and strength of the faces, smaller localized indentation damage on the bottom face was also observed in Figure 3b as compared with the other two types of tested panels with laminated paper faces only. Similar responses were observed for each replicate within the specific series.

### 3.2. Impact Analyses

#### 3.2.1. Impact Analyses of Panels with Different Configurations

**Figure 4:**
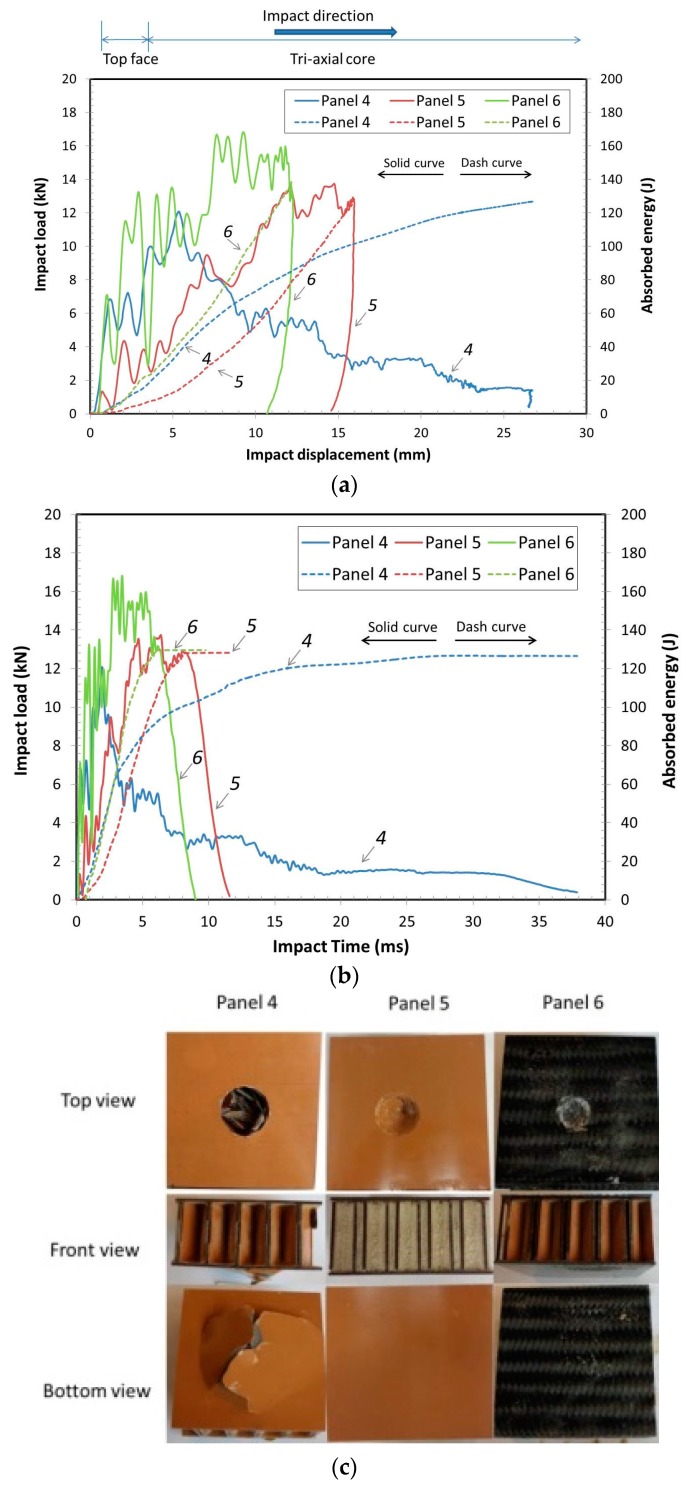
(**a**) Typical load and accumulated absorbed energy vs. displacement curves using 120 J impact energy; (**b**) Typical load and accumulated absorbed energy vs. impact time curves using 120 J impact energy; (**c**) Observed failure of three panel types after the impact test.

Typical impact energy and accumulated absorbed energy vs. impact displacement results are shown in Figure 4a from the impact load at 120 J. These showed that panel 4 made from just paper laminate had the deepest average impact penetration, 27.8 mm. The average impact peak load for panel 4 was 12.6 kN, the lowest of all the tested panels, because the stiffness and strength of the laminated paper composite was significantly less than the carbon fiber fabric reinforced faces. Panel 5, with the foam-filled core, had higher average impact peak load, 14.9 kN, with 16.7 mm average impact penetration due to the added foam’s energy absorption and its support of both the faces and the ribs. The average peak load for panel 5 was 18.3% higher than the average impact peak load of panel 4, because the foam-filled core helped to absorb more of the impact load to both core and faces, thus distributing the localized stresses and failures to more of the structure. For panel 6, it had the smallest average impact displacement, 12.8 mm, and had the highest average impact peak load of 17.9 kN. This was because the carbon fiber fabric composite face had higher stiffness and strength and toughness than either the laminated paper composite alone or with foam filled core. The carbon fiber strands radiate in three-axes causing the fiber strands to go into tension radiating out from the center of the impact load.

Figure 4b shows typical impact load and accumulated absorbed energy vs. impact time at 120 J impact energy level. The panels with either foam-filled core, panel 5, or carbon fiber fabric faces, panel 6, had shorter impact times than those for panel 4. Panel 4’s configuration resulted in further displacement over a longer period of time. They all absorbed the total 120 J of energy, but depending on how the panel was constructed resulted in slightly different absorption behavior. While panels 5 and 6 show shorter penetration times, these also resulted in higher deceleration rates or higher G’s. For some cushioning applications, a lower G deceleration would be preferred. This shows that face/core configurations can have a significant influence on engineered absorption performance and behavior.

Figure 4c shows typical failure modes for failures of each panel tested at 120 J energy. For panel 4, both faces and core were damaged. The core ribs also showed signs of buckling failure. For panel 5, while it had higher peak load, it had less of impact penetration. The foam helped absorb more of the energy and helped to prevent the ribs from breaking the bottom face. Had the impact energy been higher, then it might have been that the load head penetration would have increased sufficiently to push the ribs against the bottom face. Panel 6, with carbon fiber fabric bonded to the laminate faces and no foam showed the least damage on the top face. The carbon fibers absorbed most of the energy to prevent sufficient penetration to push the core against the bottom face and the core must have buckled slightly. These three configurations show the effects of material characteristics on impact loading for basic tri-axial core composite panels.

#### 3.2.2. Impact Analyses of Panels under Different Impact Energy

**Figure 5:**
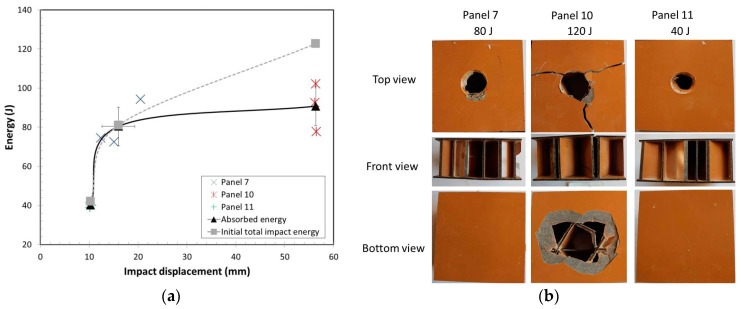
(**a**) Absorbed energy vs displacement at different initial impact energies; (**b**) Failure modes for panels at different initial impact energies.

The panels 7, 10, and 11 had the same face and core configuration but tested with impact loads of 80 J, 120 J and 40 J initial impact energy, respectively. Figure 5a shows the average of three absorbed energy vs. impact displacement with standard deviation for these panels at three different intial impact energies. The results showed that the impact energy was completely absorbed by panels 11 and 7 at 40 J and 80 J, respectively. The lower impact energy, 40 J, had a smaller displacement than for Panel 7, as expected. Both panels 7 and 11 had top face failure, shown in Figure 5b. When the initial impact energy increased to 120 J, Panel 10, the impact energy was not completely absorbed by the panel thus resulting in failure to the bottom face as the load-head to passed through the panel as shown in Figure 5b. Panel 10 absorbed an average of 91 J before the impact head passed through the panel or just 76% of its total potential energy. The impact failure test could be used to calculate the maximum absorbed energy of a panel under low-velocity impact. The load head impacted at the center area of the tri-axial core without the ribs.

#### 3.2.3. Impact Analyses of Panels with Different Impact Locations

**Figure 6:**
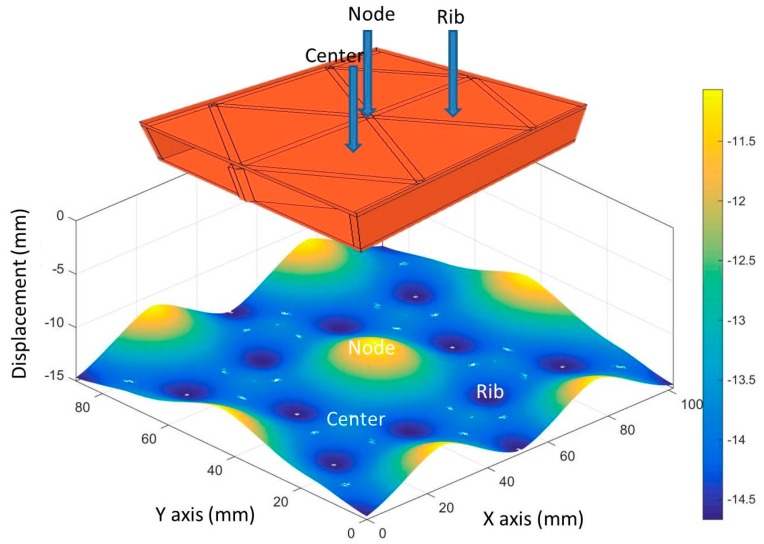
The contour of typical maximum displacement for panel at different impact locations.

Figure 6 shows a gradiant map of simulated full-field displacements at the locations based on the experimental results. The three impact displacements associated with center, mid-rib, and the rib intersection or rib-node locations were plotted. Panels 7, 8, and 9 were all impacted with 80 J of energy. These showed that the impact at the node had the smallest average displacement, 11.1 mm, followed by the center where average displacement was 13.7 mm, and the greatest average displacement was at the mid-rib with 14.7 mm. The results were expected for the rib intersection being the least displacement since the rib density was the highest at that point and provided the greatest resistance to penetration into the core. Panel 7 had the next highest average displacement where impact occurred at the center, this was initially counter-intuitive. However, after further analysis of the failure mechanism, we found that the impact head diameter was larger than the inscribed diameter between center to the ribs. The impact energy was absorbed by portions of three ribs after the impact-head broke through the face. For panel 8, the maximum average displacement, was located at the mid-point of only one rib. At impact, we believe, the one rib buckled allowing more displacement than the other two locations. 

#### 3.2.4. Impact Analyses of Panels with Different Impact Heads

**Figure 7:**
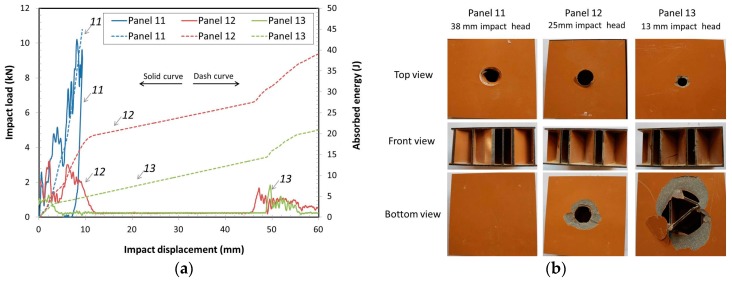
Impact energy 40 J: (**a**) Impact load and absorbed energy vs. impact displacement by different impact heads; (**b**) Failure modes of panels by different diameter impact heads.

The effects of the spherical impact-head diameter, 13 mm, 25 mm, and 38 mm were also investigated. Typical impact loads and accumulated absorbed energy vs impact displacement for the 40 J potential impact energy are shown in Figure 7a. For all the panels the impact was at the center area of the ribs. The largest impact head, 38 mm, had the highest energy absorption, 40 J, for panel 11. The results showed that the impact energy was completely absorbed by the panel. The only damage occurred on the top face and did not penetrate enough to interact with the ribs. The next smaller head, 25 mm diameter, showed that an average of 85% of the initial energy was absorbed by the panel and the damage occurred at the top and bottom faces of the panel (integrated failure). The smallest head, 13 mm diameter, had the lowest average absorbed energy, 17.3 J. Figure 7b shows the typical failure modes of the panels by different impact heads. The damage shows that the 38 mm diameter head did not pass through the top face, whereas both the 25 mm and 13 mm heads passed through both faces. For the 13 mm head, the energy lost was less on the first face than for the 25 mm head, as would be expected with the smaller head size. As the impact head diameter increased, then the absorbed energy increased as more face and core material was damaged. The lower energy loss at the top face, for the 13 mm head, translated into a higher impact speed when impacting the bottom face. Thus, the evidence for the greater bottom face fracture. As expected, energy absorption was related to impact size or area of the impactor. Energy density (J/mm^2^) was calculated based on initial energy divided by the impact area. The smallest head had an average energy density of 0.30 J/mm^2^ which passed through the laminate face. The 25 mm head had an average energy density of 0.08 J/mm^2^ and it passed through the laminate face as well. However, since the 38 mm head did not fully penetrate the top face, it had an average impact diameter of 13 mm resulting in an average resistance of 0.04 J/mm^2^. It may be possible to measure puncture resistance based on the energy density. 

#### 3.2.5. Impact Analyses of Panels with Different Size of Core

**Figure 8:**
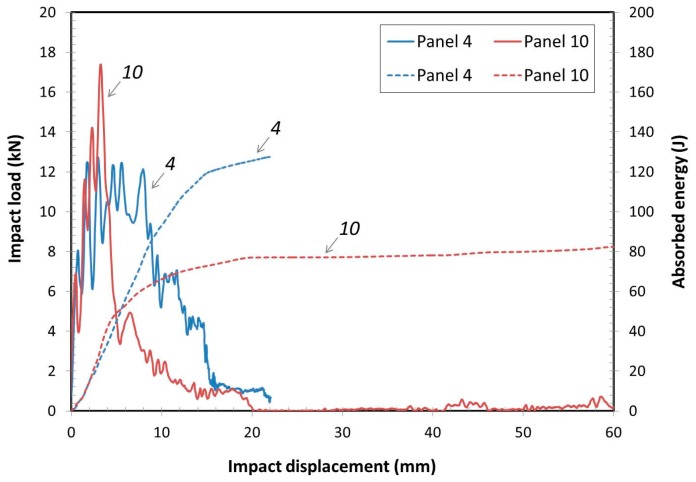
Impact load and absorbed energy vs. impact displacement for the panels with different core sizes.

The effects of core element size were investigated using a 25 mm length triangular element core and a 51 mm length triangular element core. The same thickness for the faces was used for both test panels 4 and 10. The impact load and absorbed energy vs. impact displacement for the panels with different sizes of core at 120 J initial impact energy are shown in Figure 8. The impact for both were at the center area between the tri-axial ribs. The smaller core size had higher absorbed energy capacity, because more ribs provided higher impact support resulting in higher stiffness to the faces to resist the impact load. Panel 4 had complete energy absorption with damage only on the top face. Whereas, panel 10 only absorbed and average of 74.0% of the energy in the test with failure resulting in passing through both top and bottom faces. The peak load for panel 4 was less than that of Panel 10, because the ribs in panel 4 helped to dissipate some of the impact energy. The panels with the smaller structural core had higher average impact energy absorption and less total damage to the panel. The improvement came at the expense of higher total panel weight, 38.3% higher, due to more material in the core. Obviously, design of any sandwich panel and its expected failure modes are necessary when determining construction issues.

#### 3.2.6. Comparison of Quasi-Static Compression and Impact Tests

**Figure 9:**
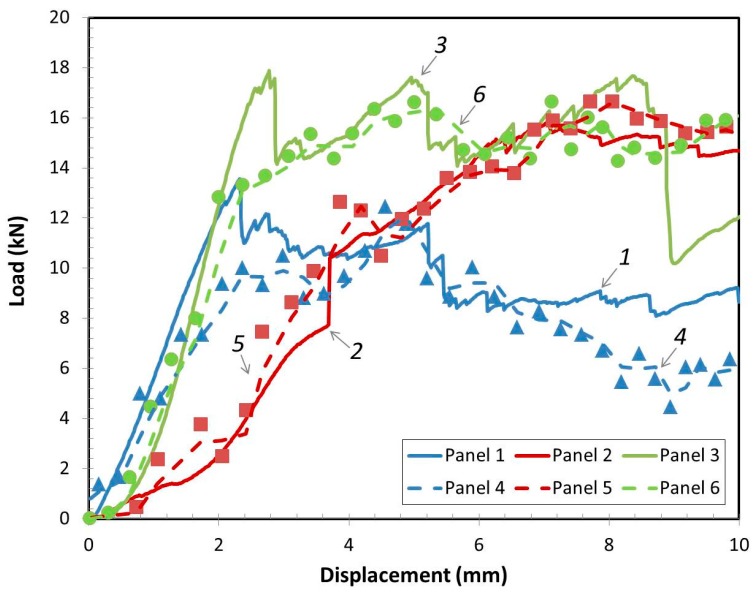
Load vs. displacement for panels with different configurations by quasi-static compression tests (solid line) and impact tests (dashed line and data points).

Load vs. displacement comparison between the quasi-static compression and impact response of the panels were analyzed using panel sets 1, 2, 3 and panel sets 4, 5, 6. Typical responses of the panels were made with three different configurations and were compared with the first 10 mm of load head contact as shown in Figure 9. The solid lines indicate the quasi-static compressive responses of panels and the dashed line and data points indicate the impact responses of the panels. The results show that for the first 10 mm of load head contact, the low-velocity impact behavior for each panel with different configurations was very similar to the quasi-static compression cases. The maximum load for each of the panels in the quasi-static compressive test had good agreement with the maximum loads of related panels for the impact tests shown in Figure 9. The carbon fiber faces panel with higher stiffness and strength properties had higher contact loads as shown in the both tests. The foam filled core decreased the load-displacement slope or helped to absorb the energy that dissipated the maximum load, which resulted in longer displacement or time domain than the panel without foam in the core. Therefore, it could be possible to correlate the ultimate compressive characteristics with similar impact behavior and use this initial result for the future design and application. 

## 4. Conclusions

Experimental investigation on bio-composite structural panels has been presented and analyzed using low-velocity impact and quasi-static compression tests. In the low-velocity impact test, the impact behavior improved when higher stiffness or strength components of panel such as carbon fiber fabric composite layer and higher triangular core density were used to support the face and core. In addition, foam filled core provided improved energy absorption and better energy transfer throughout the structure. The foam core helped to resist impact buckling in the core ribs. The impact head size influenced impact energy density. If the energy density was high enough to cause penetration through the top face, then the residual impact speed and energy density will influence the following impact on the bottom face. A smaller grid element core would provide better energy absorption due to the higher core density. 

The impact failures of composite panels could be classified into three stages according to their damage levels. The first stage is top face damage only, followed by the second stage of core buckling failure and the third stage of whole panel penetration failure. Generally, face damage only is not considered a critical failure, whereas damage that impacts the core and bottom face significantly degrades mechanical performance of any panel, thus it is not accepted for most applications. These tests show there are conditions where damage, while undesirable, could occur that only affects the top face. The tri-axial core bio-composite structure with additional modification could be shown to locally fail without significant impact on the other components. Depending on the design limits and impact needs this composite panel could be designed to meet some of these challenges. These tests also show that depending on where the impact occurs may influence the test data especially when the rib construction and impact head are similar dimensions.

Moreover, the results from compression test showed that both quasi-static compression and low-velocity impact tests had similar mechanical responses in the early phase of each test. The compression results could be used as reference to evaluate the low-velocity impact performance. For future applications, the bio-composite structural panels could be engineered to achieve various impact performance characteristics for different applications while reducing the weight and cost by optimizing the panel configurations. For further study, a micro-damage mechanism and finite element model could be used to help analyze impact performance.

## Figures and Tables

**Table 1:** Materials properties for individual structural panel components.

Materials	Nominal Thickness (mm)	Comp. Strength MD ^b^ (MPa)	Comp. Strength CD ^c^ (MPa)	Tensile Strength MD ^b^ (MPa)	Tensile Strength CD ^c^ (MPa)	MOE MD ^b^ (GPa)	MOE CD ^c^ (GPa)	Shear Modulus (GPa)	Poisson’s Ratio MD	Poisson’s Ratio CD
Laminated papers (LP)	2.4	195.1	168.7	173.9	118.6	11.6	8.3	4.3	0.36	0.27
Carbon fiber fabric w-L ^a^	3.2	195.1	168.7	216.6	132.2	16.3	13.6	6.0	0.36	0.46
Epoxy resin	-	105.9	-	31.0	-	1.4	-	0.54	0.3	-
Urethane foam	-	0.41	-	0.48	-	0.005	-	0.002	0.46	-

^a^ Carbon fiber fabric bonded with laminated paper composite; ^b^ MD: machine direction;^c^ CD: cross direction.

**Table 2:** Test method, density, core fabrication spacing and characteristics, test ball size, impact energy, and test location.

Group	Test	Panel Density (Kg/m^3^)	Core Slot Spacing (mm)	Foam	Carbon Fiber Fabric Face	Load Head Diameter (mm)	Impact Energy (J)	Impact Location
Panel 1	Quasi-static compression	455.8 (7.93) ^a^	25	-	-	38	-	center
Panel 2	Quasi-static compression	530.8 (1.83)	25	+	-	38	-	center
Panel 3	Quasi-static compression	470.9 (4.01)	25	-	+	38	-	center
Panel 4	Impact	455.8 (7.93)	25	-	-	38	120	center
Panel 5	Impact	530.8 (1.83)	25	+	-	38	120	center
Panel 6	Impact	470.9 (4.01)	25	-	+	38	120	center
Panel 7	Impact	322.3 (0.39)	51	-	-	38	80	center
Panel 8	Impact	327.6 (1.17)	51	-	-	38	80	rib
Panel 9	Impact	316.9 (1.93)	51	-	-	38	80	node
Panel 10	Impact	317.9 (1.93)	51	-	-	38	120	center
Panel 11	Impact	317.0 (5.36)	51	-	-	38	40	center
Panel 12	Impact	319.7 (1.66)	51	-	-	25	40	center
Panel 13	Impact	320.3 (1.28)	51	-	-	13	40	center

^a^ Standard error.
